# Pay-off scarcity causes evolution of risk-aversion and extreme altruism

**DOI:** 10.1038/s41598-018-34384-w

**Published:** 2018-10-30

**Authors:** R. M. L. Evans

**Affiliations:** School of Mathematics, University of Leeds, Leeds, LS2 9JT UK

## Abstract

All organisms descend from populations with limited resources, so it is clear why evolution should select strategies that win resources at the expense of competitors. Less obvious is how altruistic behaviours evolve, whereby an individual helps others despite expense to itself. Modelling simple agents using evolutionary game theory, it is shown that steady states of extreme altruism can evolve when pay-offs are very rare compared with death. In these states, agents give away most of their wealth. A new theorem for general evolutionary models shows that, when pay-offs are rare, evolution no longer selects strategies to maximize income (average pay-off), but to minimize the risk of missing-out entirely on a rare resource. Principles revealed by the model are widely applicable, where the game represents rare life-changing events: disasters or gluts.

## Introduction

Altruism exists in many species^1–4^, even microbes^5–7^. Famously, a bird’s alarm call^1^ is an altruistic trait, as it benefits others at the cost of alerting predators to the calling bird. Here (in common with refs^3,8^), we shall distinguish between “cooperation”^9^, which may benefit both parties involved, and “altruism”, which benefits *only* others, *not* the altruist.

In evolutionary game theory, the complex competitive processes of life are modelled by simple agents playing a simple game. They reproduce and die according to the game’s outcome, and offspring inherit (imperfect) copies of parents’ strategies. A number of mechanisms have been identified^10^ that can promote altruism in such models, including social compensation^11–13^, group selection^8^, repeated fragmentation into colonies^7^, or kin selection with low cost compared to conferred benefit^1,8,14,15^. These and similar mechanisms can arise spontaneously in spatially structured and fluctuating populations^4,7,16–18^. Another generic mechanism is identified in the present investigation.

Insight into altruism is gained from a standard evolutionary model, the spatial ultimatum game (UG)^19^, in which one player, the proposer, must decide how to apportion some beneficial resource between itself and the other player: the responder. If the responder accepts, the proposer keeps the remainder. If the offer is rejected, *neither* player receives anything. The proposer gains no direct benefit from the portion given away. Thus increasing that portion constitutes altruism.

The ultimatum game (UG) is a paradigm for the trading or squandering of any resource. It was originally studied in experiments on human players^19^, but the current study is not specific to humans. While simple games can successfully represent some aspects of human behaviour^4^, it should not be inferred that those same games influenced the human evolution responsible for those behaviours. Thus, when used in evolutionary models, the UG should be understood as a proxy for resource allocation during the evolution of species with simple behaviours.

The game was originally modelled^13^ in mean field — all agents interacting with all others. It has since been studied spatially^17,20,21^ and subject to noise^18,22^, yielding steady states with average offers above the Nash equilibrium (“rational” self-interest) value of zero and, in some cases^18,21^ close to the “fair” value of 50%. Higher average offers are seen in mini-game versions of the UG where only a discrete subset of strategies are allowed^23^, or if other constraints are imposed on the strategies^13,24^ or rules of the UG. See ref.^18^ for a recent review.

We shall see that, if game-play is very rare compared with death (occurring on average once in many lifetimes per individual), steady states evolve with average offers of 75% for some parameter values, without the introduction of constraints or alteration of the rules of the game.

Here (Box 1), a version of the spatial UG^20^ is used, with the order of trading and competition between agents determined stochastically (as in^22^). Their strategies evolve freely by natural selection. The model has two parameters: mutation scale *μ* and death rate *R* (equal to birth rate, and exceeding the unit gaming rate). To check model-independence of the findings, other versions with non-cumulative pay-offs, and with competitive births (rather than deaths) have also been simulated (to be published elsewhere).

### Box 1: The Model

Agents occupy a *L* × *L* square lattice, each with their own wealth *w*_*i*_ and unchanging strategy *s*_*i*_ = (*p*_*i*_, *q*_*i*_) for the UG: the offer *p*_*i*_ that it will propose, and its acceptance threshold *q*_*i*_ when responding.

**Gaming:** With unit average rate per agent (thus setting the scale for measuring time), agents are selected at random to propose their offer to one of their four nearest neighbours, chosen at random. Pay-offs awarded to the responder *j* and proposer *i* are respectively *π*_*j*_ = *p*_*i*_ and *π*_*i*_ = 1 − *p*_*i*_ if *p*_*i*_ ≥ *q*_*j*_, or *π*_*i*_ = *π*_*j*_ = 0 otherwise. The pay-off *π*_*i*_ is added to an agent’s accumulated wealth, *w*_*i*_ → *w*_*i*_ + *π*_*i*_.

**Competition:** With rate *R* per unit time per agent, agents are selected at random to reproduce asexually, producing an offspring that replaces (and thereby kills) one of its nearest neighbours. In 50% of cases, the poorest (lowest *w*) of the four neighbours is chosen, with ties between equal-poorest settled randomly. In the other 50% of cases, the neighbour is chosen entirely at random.

**Reproduction:** Offspring have no initial wealth, *w*_*o*_ = 0, and a strategy *s*_*o*_ = (*p*_*o*_, *q*_*o*_) = (*p* + *δ*_*p*_, *q* + *δ*_*q*_), deviating from the parent’s (*p*, *q*) by mutations *δ*_*p*_, *δ*_*q*_ drawn stochastically (subject to *p*_*o*_, *q*_*o*_ remaining in the interval [0, 1]) from a uniform distribution with zero mean, and standard deviation *μ*.

## Results

For the cases reported in this section, the system is initialized with the strategy (*p*_*i*_, *q*_*i*_) at each site (see Box 1) drawn independently and uniformly from the unit-square in strategy-space, *p*_*i*_, *q*_*i*_ ∈ [0, 1]. The dynamical rules are iterated while population statistics are measured as functions of time, eventually asymptoting. The asymptotic steady states were found to be independent of the initial conditions (see Methods).

### Time-scales and starting transient

**Figure 1:**
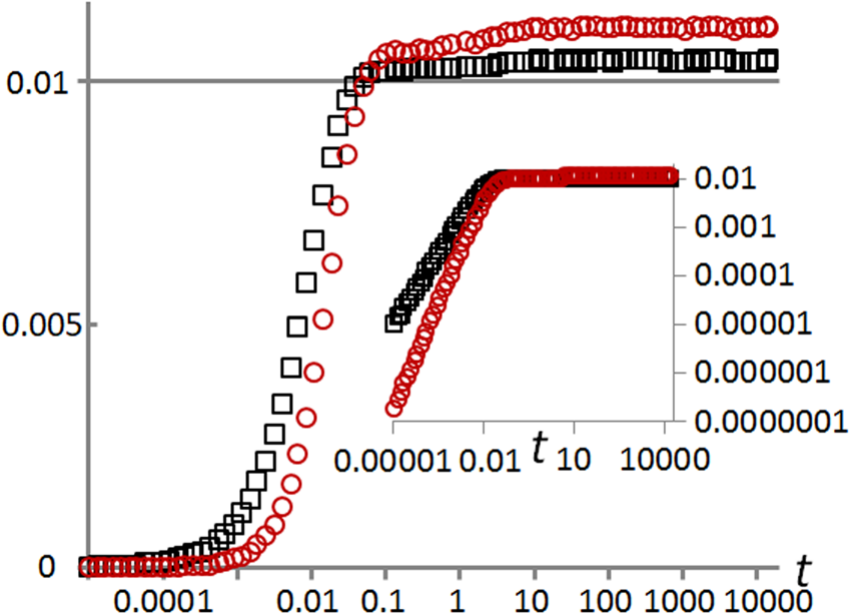
The ageing of the population is apparent at early times in this plot of the mean (black squares) and standard deviation (red circles) of the age distribution versus time (on logarithmic scale), in a simulation, with death-rate *R* = 100 and mutation strength *μ* = 0.00258. The inset shows a logarithmic age scale.

On the shortest time-scales, comparable to or smaller than *R*^−1^, the reciprocal of the death (equivalently birth) rate, the distribution of ages in the young population varies with time *t*. When $t\ll {R}^{-1}$, mean age is approximately equal to *t*. The mean and standard deviation of the age distribution are plotted against time in Fig. 1 for a typical simulation, with death-rate *R* = 100 and mutation strength *μ* = 0.00258. The age distribution remains almost constant for times $t\gg {R}^{-1}$, with just small variations visible in Fig. 1 at later times, due to changes in the distribution of strategies, which take place on longer time-scales. We next consider those strategic dynamics.

For large mutation scale $\mu \gtrsim 0.1$, strategies continue to fill the unit square, so that mean offer and acceptance values remain at $\langle p\rangle =\langle q\rangle =\frac{1}{2}$.

**Figure 2:**
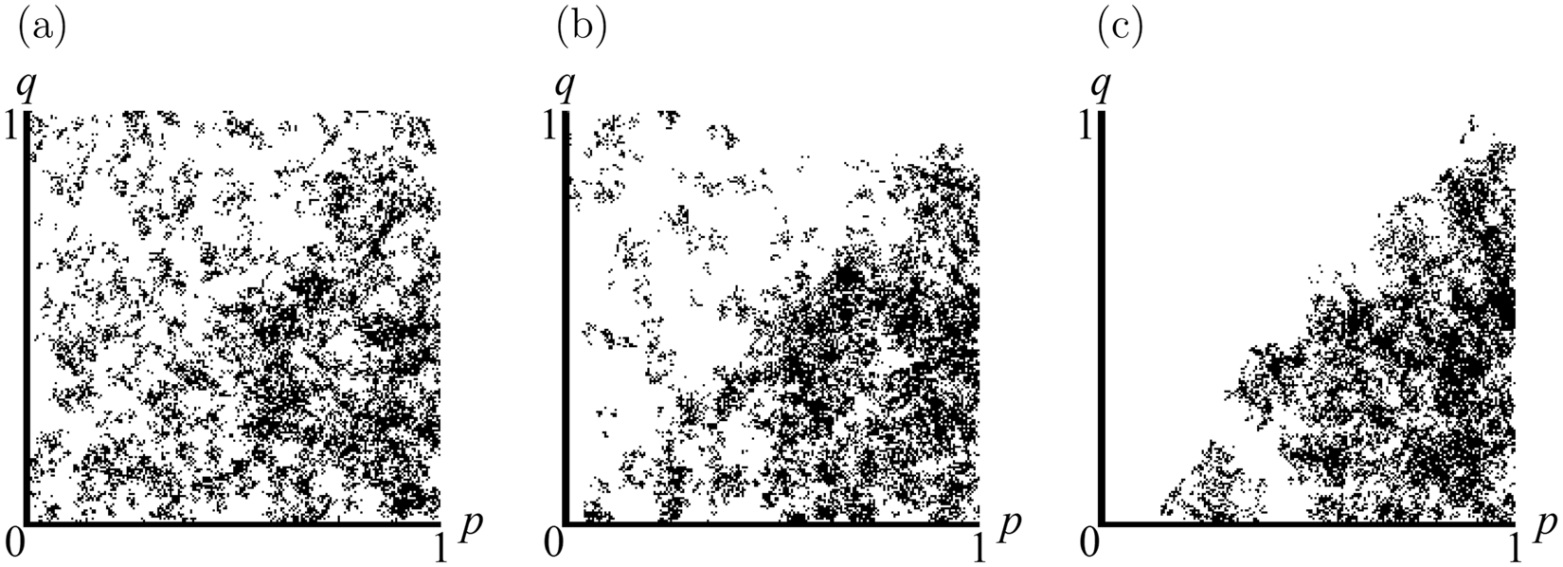
An evolving population of 16384 agents with *R* = 31, *μ* = 0.00462. Each agent’s strategy is represented by a point in the (*p*, *q*) plane, at times (**a**) *t* = 1.61, (**b**) *t* = 4.83 and (**c**) *t* = 10.32.

For small *μ* (henceforth assumed) typical intermediate-time evolution (occurring on the time-scale of the mean time between games at a site $t \sim 1$) is shown in Fig. 2. Strategies with *p*_*i*_ < *q*_*i*_ quickly die out leaving only the triangular region 0 ≤ *p* ≤ 1, 0 ≤ *q* ≤ *p* of strategy-space (henceforth called the *dominant triangle*) occupied for the remainder of the simulation. At the end of this intermediate stage, strategies fill the dominant triangle approximately uniformly, so the mean offer (at its centre of mass) is very generous, $\langle p\rangle \approx \frac{2}{3}$.

Subsequently, the distribution of strategies slowly evolves towards the final steady state, but remains confined to the dominant triangle. Within that region, generous $(p > \frac{1}{2})$ or selfish $(p < \frac{1}{2})$ strategies may dominate, depending on the parameter values *R* and *μ*.

The typical time-scale of this final approach to equilibrium is set by the diffusive motion of the population through strategy space, due to inheritance with random mutation. The typical number of generations per time-step per lattice site is given by the death (and birth) rate *R*. Each reproduction is accompanied by a mutation; a random displacement in the (*p*, *q*) strategy-space, with a variance of approximately (neglecting selection and boundaries) *μ*^2^. Hence, in the absence of selection, lineages make excursions of variance *Rμ*^2^*t* (in each direction, *p* and *q*) in time *t*. Under selection pressure, the random walks in strategy-space are modified, so that corrections to this formula arise, but it continues to provide a useful order-of-magnitude estimate.

To reach a statistically steady state, the population requires time to explore the unit-square strategy-space. That is,

1 $$t > {\tau }_{{\rm{eq}}}\equiv 1/(R{\mu }^{2}),$$

which defines *τ*_eq_, the characteristic time-scale for equilibration of the steady-state distribution of strategies that coexist in the population.

**Figure 3:**
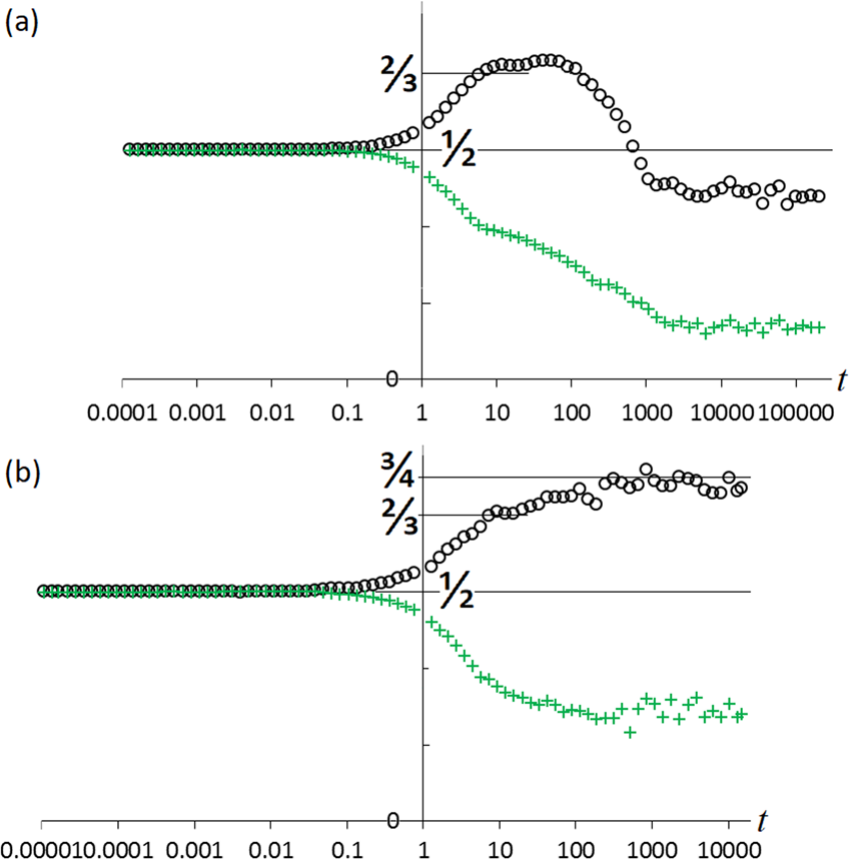
Mean offer 〈*p*〉 (black circles) and acceptance threshold 〈*q*〉 (green crosses) versus time for system size *L*^2^ = 512^2^ with (**a**) (*R*, *μ*) = (15, 0.0015), (**b**) (*R*, *μ*) = (100, 0.00258). Note transient plateaux near 5 < *t* < 50, 〈*p*〉 = 2/3 when the dominant triangle is uniformly filled. Steady-state values (with stochastic fluctuations) are reached after time *t* ≈ *τ*_eq_ = 29600 in (**a**) and 1500 in (**b**).

The evolution of the mean offer and acceptance values (〈*p*〉, 〈*q*〉), spanning all time-scales, is plotted in Fig. 3 for the cases (*R*, *μ*) = (15, 0.0015) and (100, 0.00258), for which *t*_eq_ = 29600 and *t*_eq_ = 1500 respectively.

### Steady states

**Figure 4:**
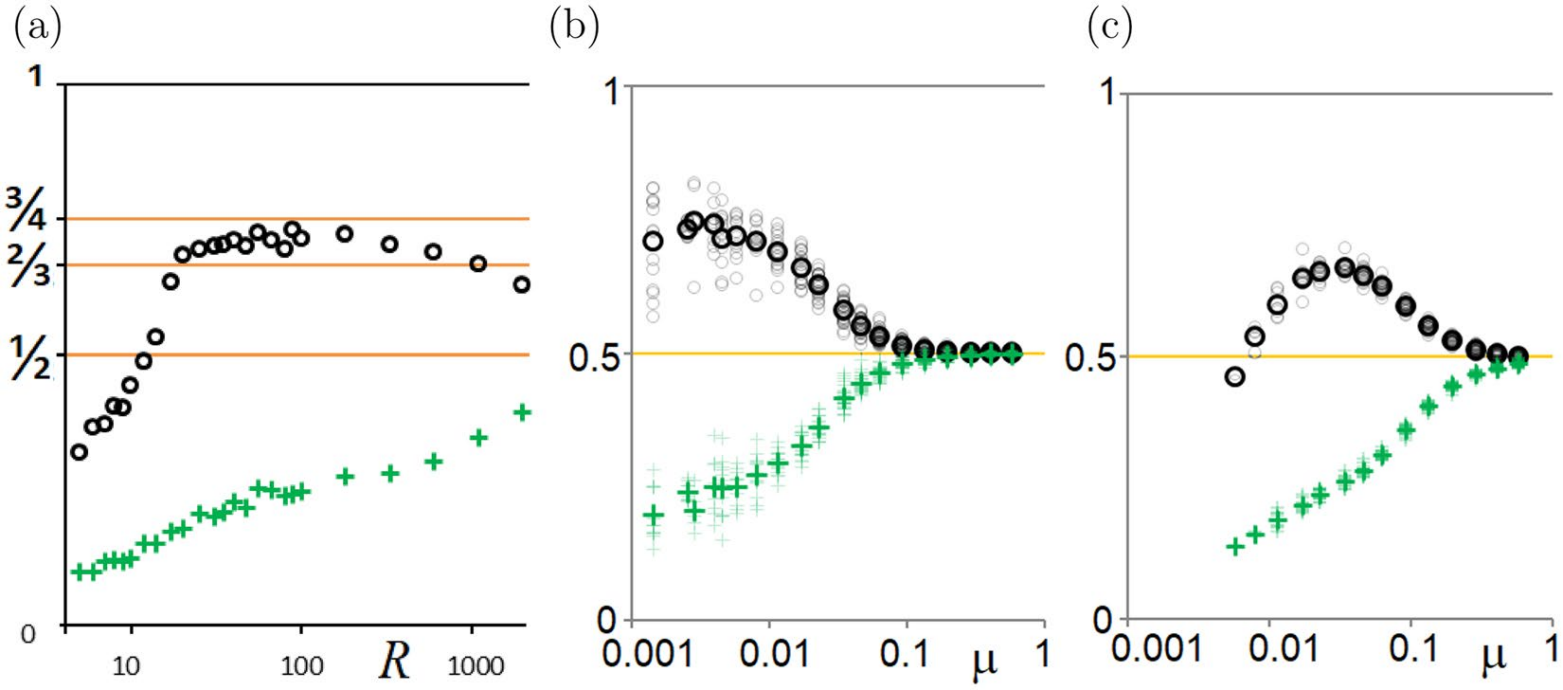
Steady-state population-averaged offer 〈*p*〉 (black or grey circles) and acceptance threshold 〈*q*〉 (green or grey crosses) versus logarithmic (**a**) death rate *R* at *μ* = 0.00462; (**b**,**c**) mutation strength *μ* at (**b**) *R* = 100; (**c**) *R* = 10. Small pale symbols are instantaneous values at different times, demonstrating fluctuations. The large bold symbols are averaged over the fluctuations.

The final statistically steady states are ascertained (see Methods) for a range of (*R*, *μ*) parameter values. Figure 4 shows the steady-state mean strategies (〈*p*〉, 〈*q*〉). At very high death rate *R*, extreme altruism (very generous offers) persists.

**Figure 5:**
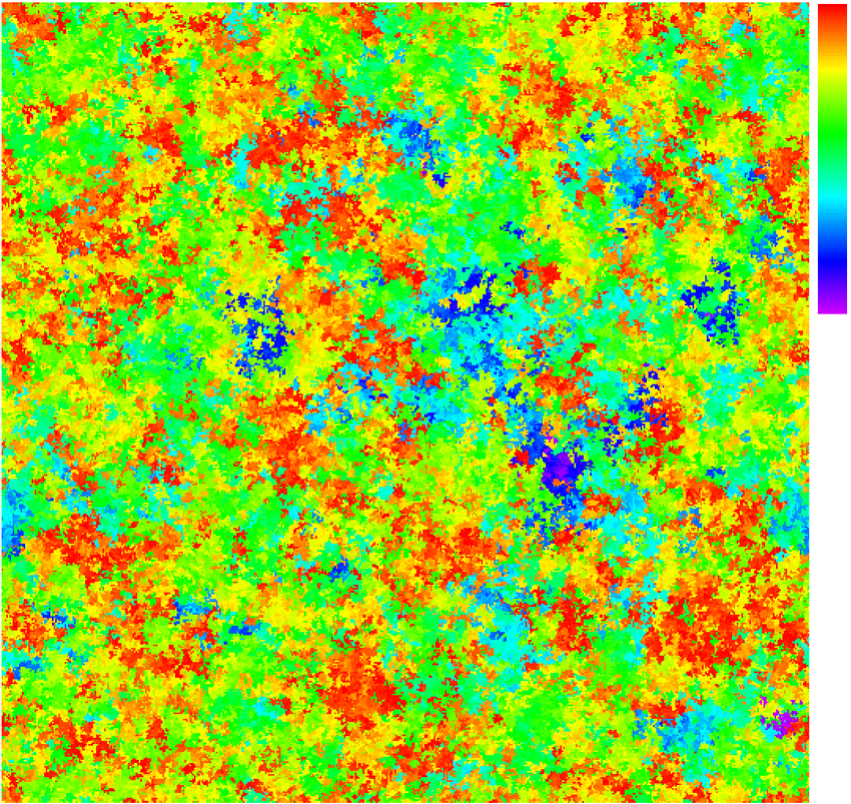
Colour-coded snap-shot of a typical spatial configuration of agents on the lattice, during the stochastic steady state of the simulation with parameters *R* = 80, *μ* = 0.00462. Each agent is coloured according to its value of *p*. Key top-right: Scale of *p* values, from *p* = 0 (violet) to *p* = 1 (red). Hence red agents give away almost all of their wealth.

A typical instantaneous configuration of agents on the 2D lattice is shown in Fig. 5 for a population in a statistically steady state at (*R*, *μ*) = (80, 0.00462), where agents are very generous on average, (〈*p*〉, 〈*q*〉) = (0.70 ± 0.02, 0.24 ± 0.01).

As we shall discuss in the next section, the reasons for the stability of the generous strategies depend on the stochastic nature of game-play. Because the order of game-play and choice of opponent is stochastic, the pay-off received for any given strategy is not uniquely determined. Instead, the pay-off for a given strategy has a distribution with a non-zero variance, so that its mean is not the only important property.

**Figure 6:**
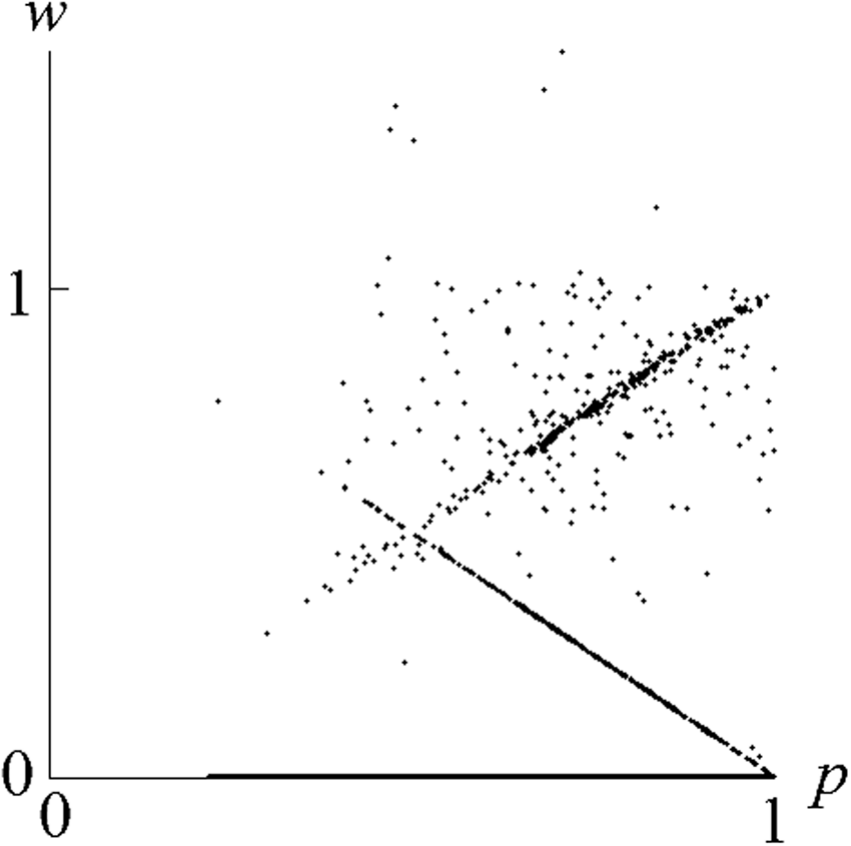
Scatter-graph of wealth *w* versus offer value *p* for all 128 × 128 agents at a typical instant, in a steady state of a simulation at death rate *R* = 80 and mutation strength *μ* = 0.00462.

This can be seen in the scatter-graph of the wealth *w* of each agent, plotted against its offer value *p*, shown for a typical steady state configuration in Fig. 6. At the death rate of *R* = 80, the UG is played at each cite typically only once in every eighty lifetimes. Hence most agents in the system have never played the ultimatum game in their lives, and therefore have no wealth, *w* = 0. They are clustered along the horizontal axis in Fig. 6. A small minority of the population has non-zero values of *w*.

Consider, for example, an agent with strategy *p* = 0.7 (and some typical value of *q*). It might or might not play the UG as proposer and/or responder and, if it does, will partner an agent with an as-yet unknown strategy. Hence the net pay-off for strategy (*p*, *q*) is not uniquely determined but (from Fig. 6 at *p* = 0.7), might be zero (most likely), or close to 0.3 or 0.7 or, with lower probability, some other value.

## Analysis

### Pay-off advantage of dominant-triangle strategies

Let us first consider why the population self-organises into the dominant triangle (Fig. 2). Neighbouring sites are usually closely related, so differ in strategy by few mutations. Hence, agents play mostly against approximately their own strategy, so effective kin selection emerges from relatedness correlations (“assortment”^3^). Thus an agent *i* is more likely to receive zero pay-off if *p*_*i*_ < *q*_*i*_. We shall next argue that such agents cannot exploit those with *p* > *q*.

### Stability against invasion

**Figure 7:**
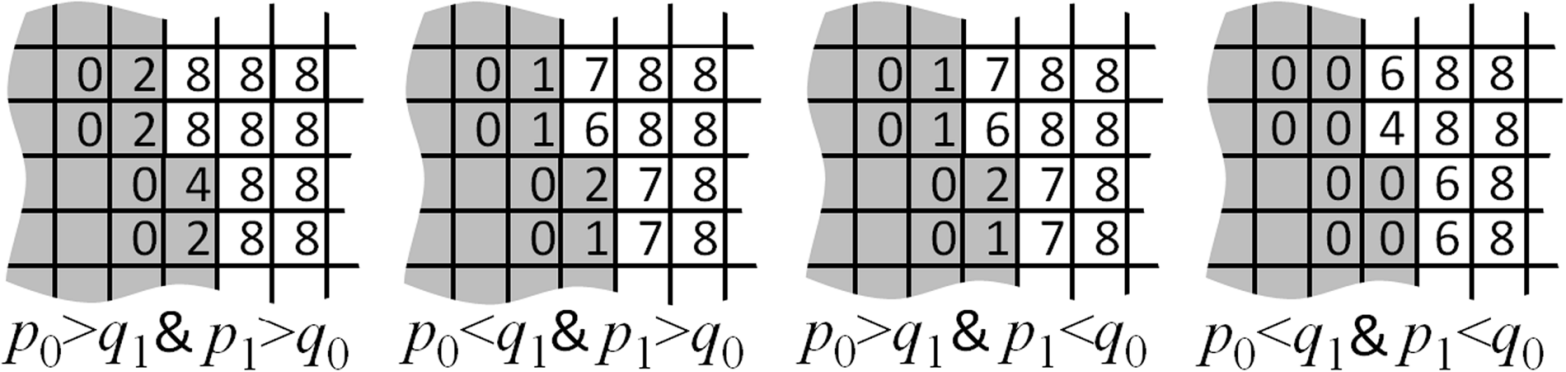
A spatial region where a local cluster of agents (grey) with strategy ≈(*p*_0_, *q*_0_); *p*_0_ < *q*_0_, meets another (unshaded) with strategy ≈(*p*_1_, *q*_1_); *p*_1_ > *q*_1_. Four cases exist. Success rates are enumerated.

Figure 7 illustrates an interface between regions of differing strategies. Within each region, agents are assumed to be locally similar. Agents shaded grey have strategies ≈(*p*_0_, *q*_0_) where *p*_0_ < *q*_0_, so reject offers from similar agents. Unshaded agents have strategies ≈(*p*_1_, *q*_1_) in the dominant triangle, *p*_1_ > *q*_1_, so accept kindred offers.

Each agent can play with four neighbours, as proposer or responder, giving it eight possible distinct games, all with equal probability. Of those eight, the number resulting in success (i.e. non-zero pay-off) is labelled for each agent. Those numbers therefore give the agents’ relative probabilities of receiving a non-zero pay-off. The pay-off magnitude remains uncertain, but irrelevant because (as we shall see) survival rate depends only on whether the pay-off is zero or non-zero, not on its mean value. The reason is that, when pay-offs are much rarer than death (high *R*), any agent with non-zero wealth is inevitably the richest in its neighbourhood, because most have never played.

So a strategy’s survival depends only on its proficiency in avoiding zero pay-off, as shown by the theorem in the next section (which is not confined to the UG). In all cases in Fig. 7, we see that unshaded agents have higher success rate, so out-compete those (shaded) outside the dominant triangle.

Away from the interface, similar strategies have identical success rate hence, by the theorem below, equal fitness. So lineages diffuse through a flat fitness landscape in the dominant triangle, filling it uniformly. This explains the approximate result 〈*p*〉 ≈ 2/3.

If we relax the assumption of locally similar strategies, and consider instead the opposite limit; well mixed agents; then a generous population again remains stochastically stable^25^ by virtue of the high-death-rate theorem (in the next section). In that case, a population of agents with a distribution of strategies uniformly filling the region (*p* > 1/2; *q* < 1/2) all have maximum success rate (no offers rejected), because *p* > *q* in every case. In the presence of such a population, a cheat with *p* < 1/2 would have a higher pay-off whenever its offer is accepted, but a lower probability of acceptance (and thus of non-zero pay-off), and hence a lower fitness, by the theorem. So the cheat cannot invade.

Such a population has mean 〈*p*〉 = 3/4. There is some evidence of states close to this in Figs 3b and 4a.

## Theorem: Evolution at high death rate

Much of the behaviour discussed above is attributable to a general feature of evolution at high death-rate (compared with the rate of game play), not confined to a specific evolutionary game.

All agent-based evolutionary models include two processes:

(i) Agents are assigned wealth depending on their strategies and on some rules of game-play, which may have a stochastic element arising from the game itself or from the order of play or from the environment of each agent and its neighbours.
(ii) Agents are replaced/reproduced according to some rules that depend on their wealth. This stage involves comparing the wealths of some local neighbourhood of *z* competing agents (where *z* → ∞ would be the well-mixed mean-field case). In particular, when death is wealth-dependent then, in competitions amongst a local neighbourhood of *z* agents, one is killed. Survival probability for each agent is some function *K*(*w*, {*w*_*i*_}) of its wealth *w* and those {*w*_*i*_} of competing agents. (An example, defined by the competition in Box 1 in the absence of any ties, would be $K=\tfrac{1}{2}\mathrm{[1}-1/z]$ if $w\, < \,{w}_{i}\,\forall \,i$ and $K\,=\,1-1/2z\,{\rm{i}}{\rm{f}}\,w\, > \,{w}_{i}$ for some *i*).

Let us consider some general properties of *K*(*w*, {*w*_*i*_}).

- In any neighbourhood, the agents’ probabilities of *not* surviving must sum to unity, because exactly one agent will be killed. That is,
  2 $$\sum _{j}^{z}\,\mathrm{[1}-K({w}_{j},\{{w}_{i}:i\ne j\})]=1.$$
- In the special case of a neighbourhood in which all *z* of the agents have equal wealth *a*, all must have equal survival probability. Hence, from Eq. 2,
  3 $$K(a,\{a,a,\ldots \})=1-1/z.$$
- In the special case where all agents have equal wealth *a* except for one with wealth *w*, from Eq. 2, we have
  4 $$1-K(w,\{a,a,a\ldots \})+(z-\mathrm{1)}(1-K(a,\{w,a,a\ldots \}))=1$$
  because their probabilities of *not* surviving must sum to unity (one agent will be killed).
- In any reasonable model, *K*(*w*, {*w*_*i*_}) is a non-decreasing function of *w*, because being richer is never a disadvantage.
- Some updating schemes have an in-built wealth scale defining the rate at which survival probability *K* rises with increasing wealth *w*. For instance, in the “smoothed imitation” scheme^26^, the probability of an agent with wealth *w*_*a*_ replacing one with wealth *w*_*b*_ is proportional to 1/{1 + exp[(*w*_*b*_ − *w*_*a*_)/*α*]} with *α* defining a wealth scale. Many other common updating schemes are *scale-free*, so that the relative importance of different wealths is determined only by those values present in the neighbourhood. Examples include the “imitate if better” rule^26,27^, the “replicator rule”^17,28,29^ and ranked schemes where survival depends only on the *order* of wealth (wealthiest to poorest), as well as the linear scheme ($K=\lambda w/(w+\sum \,{w}_{i})$), and also the scheme in Box 1. For scale-free schemes, in the special case where all agents in a neighbourhood have zero wealth except for one with wealth *w* > 0, its survival probability is independent of the magnitude of *w*, that is

5 $$K(w,\{0,0,0\ldots \})=\lambda $$

where *λ* is a constant.

We shall consider the case where the above processes (i) and (ii) do not involve the same sets of agents; i.e. each agent does not compete for survival with the same agents with which is has played the game. This is usually the case since, in a well-mixed population, the same pair of agents is unlikely to meet twice. And, on a square lattice, games are played by nearest neighbours while competition is between next-nearest neighbours (the four neighbours of a focal agent that will replace one of them). In this case, the wealths *w* and {*w*_*i*_} of competing agents are not the result of the same instance of game-play, and hence are correlated with each other only weakly, via the correlations between strategies in the neighbourhood.

Consider a large, structured population of agents, with a variety of strategies and wealths, subject to the processes (i) and (ii) and the conditions specified above. Within that population, let us consider only that subset of agents that have a particular strategy *s*. Those agents have various values of wealth *w* (as illustrated by a scatter of points at fixed *p* in Fig. 6), with some emergent distribution *f*_*s*_(*w*). (A mean-field analysis would use only the strategy-dependent mean wealth 〈*w*〉_*s*_, instead of the full distribution).

Next, let us consider all those agents that can compete against any agent with strategy *s*, because they belong to the same neighbourhood. Those competitors against strategy *s* have various values of wealth *w*, with some emergent distribution *g*_*s*_(*w*).

Given that survival probability is *K*(*w*, {*w*_*i*_}) and that the wealths of an agent with strategy *s* and its competitors are drawn independently (by the assumption below Eq. 5) from the distributions *f*_*s*_(*w*) and *g*_*s*_(*w*) respectively, the survival rate of strategy *s* is

6 $$P(s)=\sum _{w,\{{w}_{i}\}}\,K(w,\{{w}_{i}\}){f}_{s}(w)\,\prod _{j=1}^{z-1}\,{g}_{s}({w}_{j}),$$

Some agents have exactly zero wealth, because they have either never played the game or played it unsuccessfully. Let us define the total probabilities of *any* non-zero wealth for an agent with strategy *s* and its competitor as *ε*_*s*_ and *ε*′_*s*_, respectively, and ${\hat{f}}_{s}$ and ${\hat{g}}_{s}$ as normalized distributions of non-zero wealth, such that ${\hat{f}}_{s}$(0) = ${\hat{g}}_{s}$(0) ≡ 0. Then

7 $${f}_{s}(w)=\mathrm{[1}-{\varepsilon }_{s}]\,\delta (w)+{\varepsilon }_{s}{\hat{f}}_{s}(w),$$

8 $${g}_{s}(w)=\mathrm{[1}-{\varepsilon ^{\prime} }_{s}]\,\delta (w)+{\varepsilon ^{\prime} }_{s}{\hat{g}}_{s}(w\mathrm{).}$$

If *w* takes discrete values, *δ*(*w*) is the Kroenecker delta *δ*_*w*,0_. For continuous *w*, *δ*(*w*) is the Dirac delta and all summations are read as integrations.

If non-zero pay-offs are rare then *ε*_*s*_ and *ε*′_*s*_ are small, so, substituting Eqs 7 and 8, into Eq. 6 and expanding to first order gives,

9 $$\begin{array}{rcl}P(s) & = & [1-{\varepsilon }_{s}-(z-1){\varepsilon ^{\prime} }_{s}]\,K(0,\{0\ldots \})+{\varepsilon }_{s}\,\sum _{w}\,\hat{f}(w;s)\,K(w,\{0\ldots \})\\  &  & +(z-1){\varepsilon ^{\prime} }_{s}\sum _{w}{\hat{g}}_{s}(w)\,K(0,\{w,0,0\ldots \}).\end{array}$$

Now, substituting from Eqs 3 and 4 with *a* = 0 gives

10 $$P(s)=(1-\frac{1}{z})(1+{\varepsilon ^{\prime} }_{s}-{\varepsilon }_{s})+\sum _{w > 0}\,K(w,\{0\})\,[{\varepsilon }_{s}{\hat{f}}_{s}(w)-{\varepsilon ^{\prime} }_{s}{\hat{g}}_{s}(w)].$$

Irrespective of the selection rule (characterized by function *K*), non-zero wealth (*w* > 0) is favourable, so 1 − 1/*z* ≤ *K*(*w*, {0}) ≤ 1. Hence for any model, *P*(*s*) lies between *P*_1_ = 1 − 1/*z* and *P*_2_ = 1 − 1/*z* + (*ε*_*s*_ − *ε*′_*s*_)/*z* to first order in *ε*_*s*_ and *ε*′_*s*_. Finally, from Eq. 5 for scale-free updating schemes we have

11 $$P(s)=1-1/z+({\varepsilon }_{s}-{\varepsilon ^{\prime} }_{s})(\lambda -1+1/z),$$

which depends on *ε*_*s*_ and *ε*′_*s*_ but not on ${\hat{f}}_{s}$ or ${\hat{g}}_{s}$.

So leading-order dependence of survival probability on strategy is independent of any features of the strategy-dependant wealth distribution *f*_*s*_(*w*) (e.g. its mean) except the total probability *ε*_*s*_ of *any* non-zero wealth. Thus, strategies that yield a higher average pay-off 〈*w*〉_*s*_ carry no benefit (to dominant order) and will actually be suppressed if they enhance, even by a little, the risk (1 − *ε*_*s*_) of zero pay-off.

## Discussion and Conclusions

In summary, two different but related results have been demonstrated. The first is very general — that, when pay-offs are rare, strategies evolve to minimize the risk of receiving no pay-off, instead of maximizing revenue. Any strategy that enhances the risk is suppressed, irrespective of its *average* pay-off.

The second result follows from applying that general principle to the UG. At high death rate (compared to gaming rate), a strategy of greed cannot prevail in the UG, even though it enhances the mean pay-off (because of the possibility of a big win), because it also increases the risk of receiving nothing.

The reason is that, when pay-offs are rare, most agents have never played the game in their lives, so have zero wealth (the amount they were born with). Hence any agent that has a non-zero pay-off is wealthier than its neighbours. Increasing that pay-off would have no effect - they would still be the wealthiest.

In the UG, this risk-minimization creates a flat fitness landscape (since success rate in Fig. 7 is independent of *p* within the dominant triangle), favouring altruism in stochastic steady states. Thus most agents carry a predisposition for generosity, without ever encountering an opportunity to exercise it (gaming being rare).

Real-world applications are ubiquitous if the game represents rare life-changing events (disasters or gluts) requiring decisive action to avoid losing out. Altruistic traits thus engendered could meanwhile manifest in small acts of generosity with lesser consequences for reproductive success.

These results might be tested experimentally if very many generations of a microbial population are cultured whilst, at very low rate-density, pairs of individuals are given the opportunity to share a highly beneficial resource. Such a scenario presents a significant experimental challenge.

## Methods

All simulations were performed on a two-dimensional square lattice of *L*^2^ sites with periodic boundary conditions. For a representative sample of (*R*, *μ*) parameter values, calibration was performed by varying the system size *L*, the duration *t* of simulations and the initial conditions, and observing the effects on a set of statistical properties of the system: the mean and standard deviation of the age distribution, of the wealth distribution, of the acceptance thresholds, and the first four cumulants of the offer distribution.

**Figure 8:**
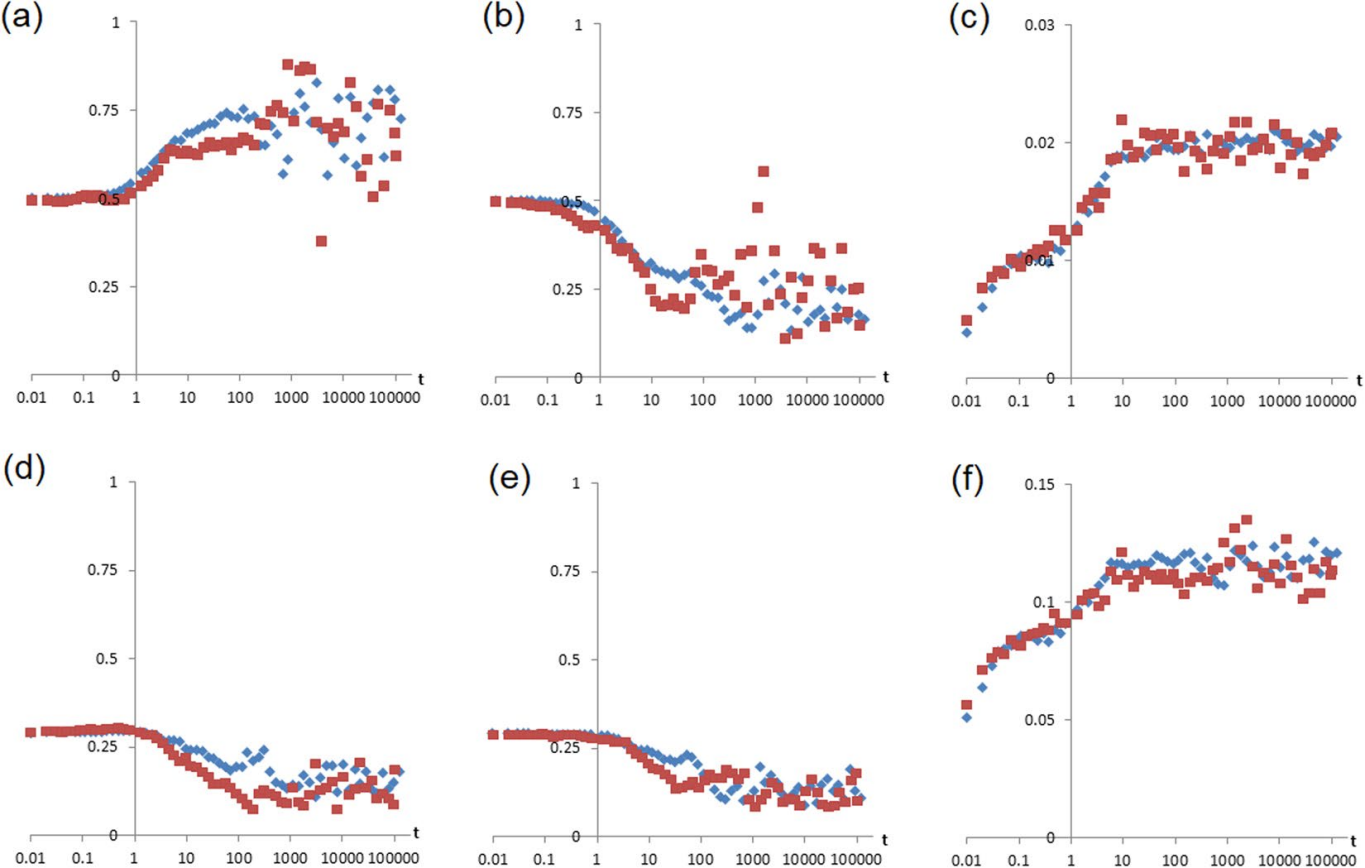
Examples of data taken from different sizes of simulations with (*Rμ*) = (100, 0.00144), to check that results are size-invariant. (**a**) Mean offer 〈*p*〉, (**b**) mean acceptance 〈*q*〉, (**c**) mean wealth 〈*w*〉, (**d**) standard deviation of offers, (**e**) standard deviation of acceptance, (**f**) standard deviation of wealth, as functions of time (logarithmic horizontal scale) for *L* = 128 (red squares) and *L* = 256) (blue diamonds).

To avoid finite-size effects, the system size *L* was increased until all results became independent of *L*. Some of the calibration data are shown in Fig. 8. Results presented above are for sizes ranging from *L*^2^ = 128^2^ to 512^2^ = 262144 sites.

To establish that the long-time limit (the steady-state) had been reached, for all cases reported in Fig. 4, asymptoting of all statistics was checked in every case by analysing the time-dependence of the full set of calibration statistics. By using a logarithmic time axis (as, e.g. in Figs 1 and 3), relaxation processes were observed, some of them on very long time-scales (as discussed above). All simulations were run for at least one order of magnitude beyond the time-scales of any systematic change in the measurements. It is perhaps of interest to note that, for the randomized initializations, the time taken to asymptote was always less than (but of the order of) *τ*_eq_ defined in Eq. 1.

Furthermore, the steady-state results were established to be independent of the initial conditions for a representative subset of the simulated parameter sets (including the full range of parameters used in Fig. 4b) by comparing the late-time results following two very different initial conditions. For a large system with a continuous set of strategies, it is impossible to establish unequivocally the absolute stability of the late-time state, since that would require an infinite set of initial conditions to be tested, and each simulated until *t* → ∞. It is therefore necessary to be selective in the initializations employed, but important (as noted in ref.^30^) to use more than one.

One of the initializations, already discussed, was fully randomized, with the values of *p* and *q* at each site drawn independently from a uniform distribution in the interval [0, 1]. This initialization was chosen as it contains no preconceptions about the possible final states.

**Figure 9:**
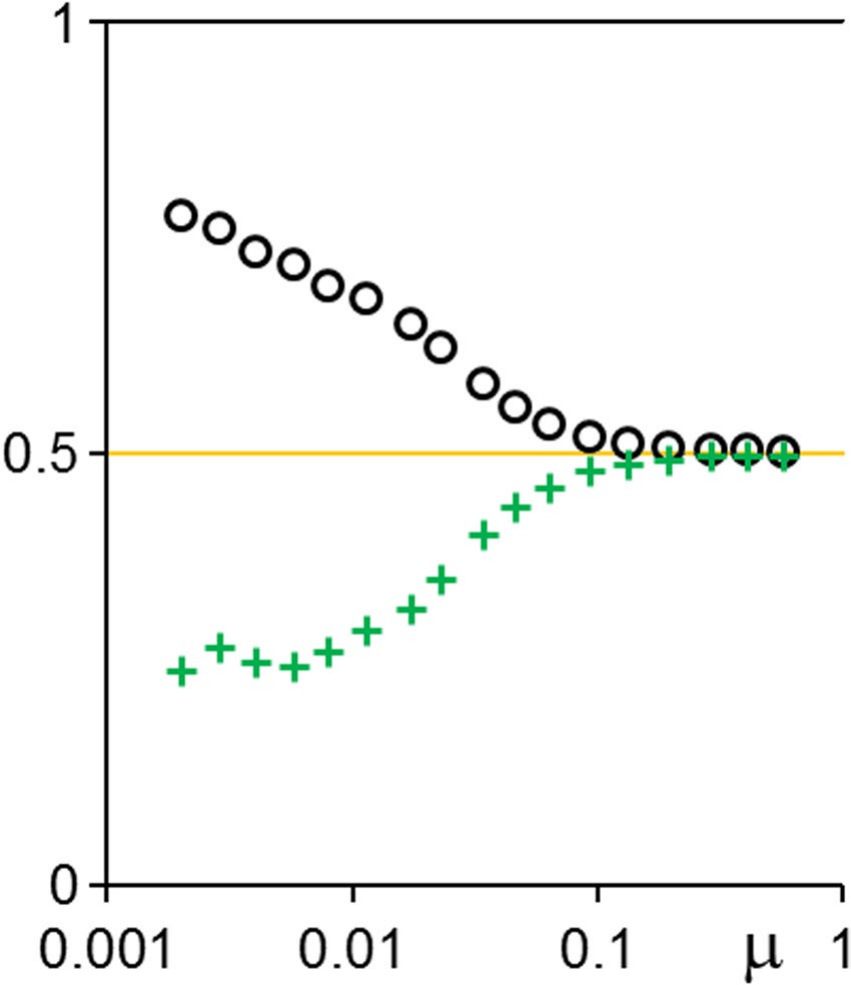
Steady-state mean offer 〈*p*〉 (black circles) and acceptance threshold 〈*q*〉 (green crosses) versus logarithmic mutation strength *μ* at *R* = 100, following a half-and-half initialization.

In the other initialization, the archetypal selfish and generous states each filled half of the lattice, meeting at a straight interface down the middle of the lattice and another at the *x* = 0 periodic boundary. In the selfish half, (*p*, *q*) = 0 for every agent. In the generous half, *p* = 1 while *q* is drawn randomly from a uniform distribution in the interval [0, 1] for each agent. Hence, all agents, on both sides of the interface, have strategies within the dominant triangle. This half-and-half initialization is designed both to be very different from the randomized initialization, and to overcome metastability near any phase transitions between selfish and generous states. Figure 9 shows late-time results following a half-and-half initialization, yielding results consistent with Fig. 4b where the randomized initialization was used.

**Figure 10:**
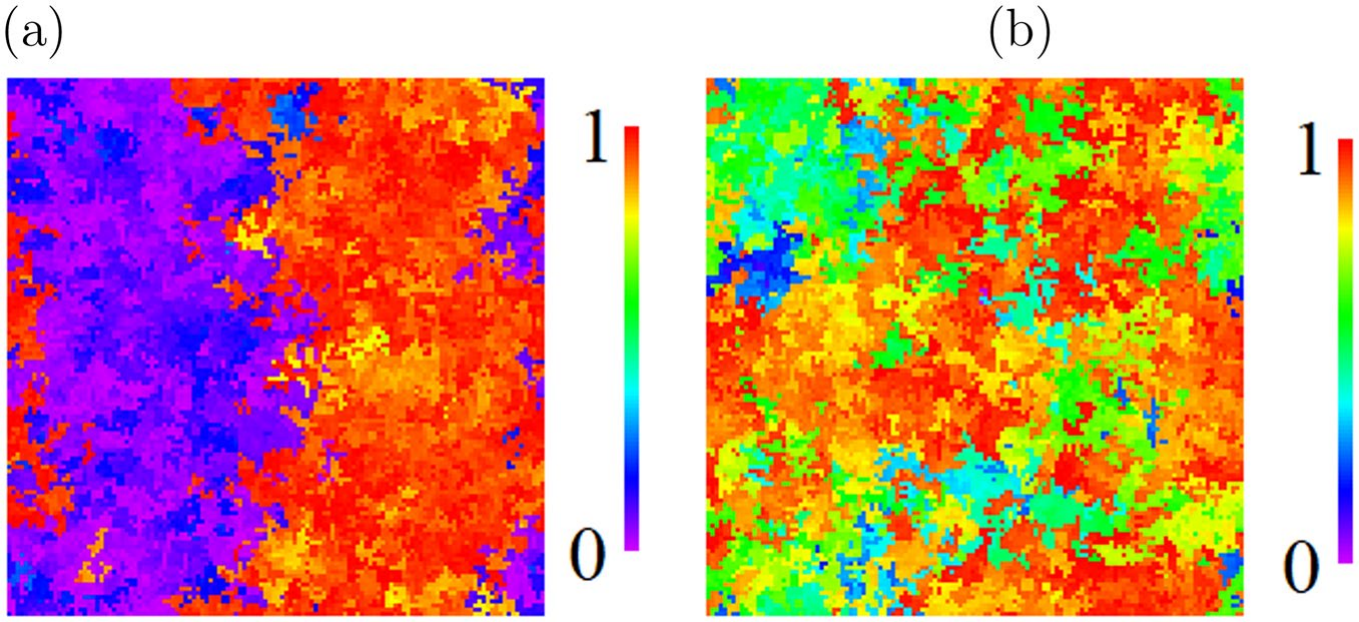
Typical instantaneous spatial configurations on a 128 × 128 lattice for *R* = 80, *μ* = 0.00462, following a half-and-half initialization, with the left-half of the system extremely selfish (*p*, *q*) = (0, 0) and the right-half extremely generous (*p* = 1, *q* ∈ [0, 1]). Each site is colour-coded by the agent’s strategic *p* value (the offer that it always makes), with values shown on the colour-scale. Snapshot are shown (**a**) at *t* ≈ 3 following an initialization, (**b**) at steady state long after initialization. Boundary conditions are periodic.

Snap-shots of a system following a half-and-half initialization are shown in Fig. 10, for comparison with Fig. 5, which followed a randomized initialization at the same parameter values (and a larger system).

These initialization protocols are similar to the “stability of subsystem solutions” procedure introduced by Perc^30^ for models with discrete sets of strategies and no mutation. However, in the present study, the selfish and generous phases are not individually time-stepped prior to being brought into contact because (a) the time-scale allowed for such a procedure must anyway remain arbitrary, (b) if the system size is sufficient, each phase will equilibrate locally before the other phase can significantly invade (c) all simulations here are run until fully equlibrated (asymptoted), instead of observing only the initial direction of interfacial movement (which could be non-monotonic).

Uncertainties *σ*_*M*_ quoted in the Results are one standard deviation of the mean, rounded to one significant figure. That is ${\sigma }_{M}=\sigma /\sqrt{N-1}$ where *σ* is the standard deviation of a sample of *N* independent values, sampled for different random number seeds and/or different times separated by a duration of at least the diffusive relaxation time *τ*_eq_.

### Code Availability

The simulation code used to generate data for this study is available at https://github.com/RMLEvans/UltimatumGame.

## Data Availability

Data that support the findings of this study, including the data presented in Figs 1, 3, 4, 8 and 9 are available in the Research Data Leeds repository at 10.5518/458.
